# Diagnostic Accuracy of Clinical Examination for Carpal Tunnel Syndrome: Validation Using Nerve Conduction Studies

**DOI:** 10.7759/cureus.87563

**Published:** 2025-07-08

**Authors:** Arsalan Shah Roghani, Jawad Farooq, Waheed Ullah, Faizan Shah Roghani, Bilal Ahmad, Atizaz A Jan, Nouman Siddique, Farhan Shahzad

**Affiliations:** 1 Trauma and Orthopaedics, Medical Teaching Institution (MTI) Khyber Teaching Hospital, Peshawar, PAK; 2 Emergency Medicine, Medical Teaching Institution (MTI) Khalifa Gul Nawaz Teaching Hospital, Bannu, PAK; 3 General Surgery, Medical Teaching Institution (MTI) Hayatabad Medical Complex, Peshawar, PAK; 4 Trauma and Orthopaedics, Saidu Group of Teaching Hospitals, Swat, PAK; 5 Internal Medicine, Medicent Hospital, Islamabad, PAK; 6 Internal Medicine, Saidu Group of Teaching Hospitals, Swat, PAK

**Keywords:** carpal tunnel syndrome, diagnostic accuracy, nerve conduction study, phalen's maneuver, tinel's sign

## Abstract

Background

Carpal tunnel syndrome (CTS) is a type of compression of the median nerve at the wrist that results in sensations of numbness, tingling, and pain in the hand. Early and accurate diagnosis is important to manage the condition and prevent irreversible nerve damage. Clinical examination is usually the initial tool used in diagnosing CTS; however, nerve conduction studies (NCS) are considered the most definitive. The purpose of this research is to evaluate and determine the sensitivity and specificity of clinical examination in diagnosing CTS, using NCS as the reference standard, among patients attending Khyber Teaching Hospital, Peshawar, Pakistan.

Objectives

The objective of this study is to assess the diagnostic accuracy of clinical examination, including Tinel’s sign and Phalen’s maneuver, for CTS, using NCS as the reference standard, among patients at Khyber Teaching Hospital, Peshawar.

Study design

This is a cross-sectional study.

Place and duration of study

Data was collected in the Department of Trauma and Orthopedics, Khyber Teaching Hospital, Peshawar, from September 1, 2024, to March 31, 2025.

Materials and methods

This cross-sectional study involved 100 patients presenting with features suggestive of CTS at Khyber Teaching Hospital, Peshawar. Patients were evaluated using both clinical examinations and NCS. Clinical examination included assessment of symptoms (such as pain, numbness, and tingling) and signs (such as Tinel's sign and Phalen's maneuver). NCS measured median nerve conduction velocity and amplitude, with abnormal findings confirming the diagnosis of CTS. The SPSS version 24 was used to analyze the results by applying the Chi-square test, which were then presented in descriptive and tabular form.

Results

In our study at Khyber Teaching Hospital, Peshawar, clinical examination for CTS showed a sensitivity of 75% and a specificity of 60% compared to NCS. The positive predictive value (PPV) was 70%, indicating that 70% of patients clinically diagnosed with CTS were confirmed by NCS. The negative predictive value (NPV) was 65%, suggesting that 65% of patients without clinical signs had normal NCS results. The calculated p-value of 0.045 indicated a statistically significant correlation between clinical examination findings and NCS results. These findings underscore the importance of using NCS as a confirmatory test following clinical suspicion of CTS for accurate diagnosis.

Conclusion

Clinical examinations (such as Tinel's sign and Phalen's maneuver) are useful but should be supplemented with NCS for accurate diagnosis and effective management planning of CTS in clinical practice.

## Introduction

Carpal tunnel syndrome (CTS) is one of the most prevalent types of entrapment mononeuropathy, affecting the median nerve as it travels through the carpal tunnel located in the wrist. It is characterized by tingling, numbness, pain, and weakness of the hand, particularly in the thumb, index, and middle fingers [1]. Clinically certain CTS was present in 3.8% of the population; the frequency of electrophysiological median neuropathy and median nerve symptoms was 4.9%, while the prevalence of CTS that was both clinically and electrophysiologically verified was 2.7% [2]. CTS is more likely to occur in certain populations, such as frequent wrist users, pregnant women, and patients with diabetes or rheumatoid arthritis. The diagnosis of CTS is clinical and involves the evaluation of the patient’s symptoms and signs, which may include Tinel’s sign and Phalen’s maneuver [3]. Tinel’s sign is elicited by tapping over the median nerve to induce tingling in the fingers, while Phalen’s maneuver involves wrist flexion to provoke symptoms. Although these clinical tests are helpful, they are not highly reliable for diagnosis; there is a notable possibility of either missing the condition or misdiagnosing it [4,5]. Studies comparing clinical signs like Phalen’s and Tinel’s to nerve conduction studies (NCS) report wide variability in diagnostic performance [6,7]. As stated earlier, NCS is considered the most reliable diagnostic tool for CTS. These studies quantify the conduction velocity and amplitude of the median nerve, providing objective evidence of nerve compression [8]. However, the use of NCS is often limited by resource constraints, and these tests are not always available at the time of initial presentation. Therefore, clinical examination remains the primary diagnostic approach in patients with peripheral neuropathy in resource-limited settings [9]. The purpose of this study is to compare the diagnostic efficiency of clinical examination with that of NCS in diagnosing CTS among patients attending Khyber Teaching Hospital, Peshawar. By evaluating sensitivity, specificity, positive predictive value (PPV), and negative predictive value (NPV), this study aims to assess the diagnostic accuracy of clinical examination, including Tinel’s sign and Phalen’s maneuver, for CTS, using NCS as the reference standard. The outcome of this study will be useful in clinical practice, particularly regarding the use of NCS in diagnosing CTS and managing patients who present with symptoms of the condition, especially in resource-limited settings.

## Materials and methods

This cross-sectional study was conducted at the Department of Trauma and Orthopedics, Khyber Teaching Hospital, Peshawar, from September 1, 2024, to March 31, 2025, after obtaining ethical approval from the hospital’s ethical committee. The study included adult patients aged 18 and above with clinical signs suggestive of CTS, such as nocturnal hand numbness, tingling in the distribution of the median nerve, and hand weakness. Patients with a prior history of CTS surgery, diabetes-related polyneuropathy, or cervical radiculopathy were excluded.

The sample comprised 100 patients with clinical features suggestive of CTS. The patients’ mean age was 45 ± 12 years, with a male-to-female ratio of 40:60. Most patients had experienced CTS symptoms for approximately 6 ± 2 months.

After obtaining informed consent, participants underwent a structured clinical evaluation, which included symptom assessment (pain, numbness, tingling) and physical examination focusing on Tinel’s sign and Phalen’s maneuver. NCS were then performed by qualified technicians using standardized protocols (e.g., patient lying comfortably, removal of metal jewelry, and maintaining normal skin temperature) to assess median nerve conduction velocity and amplitude. Abnormal findings confirmed the diagnosis of CTS.

Demographic information, as well as clinical and neurological examination findings, were documented along with the NCS results.

Data analysis was performed using the SPSS version 24. Age and other continuous variables were reported as mean ± SD. The results of the clinical examination were compared with NCS findings, and sensitivity, specificity, PPV, and NPV were calculated. A Chi-square test was applied to assess the association between clinical examination findings and NCS results, and the outcomes were summarized with the corresponding p-value. Statistical significance was set at p < 0.05.

## Results

The demographic data and clinical examination outcomes are presented in Tables 1-2, respectively. For the 100 patients investigated, clinical examination had a sensitivity of 75% and a specificity of 60% compared to NCS. The PPV was 70%, meaning that 70 patients with clinically diagnosed CTS had positive NCS results. The NPV was 65%, indicating that 65 patients without clinical signs had normal NCS outcomes (Table 3).

**Table 1:** Patient demographics. M/F: Male-to-Female ratio.

Demographic Variable	Value
Total patients	100
Age (mean ± SD)	45 ± 12 years
Gender (M/F)	40 / 60
Duration of symptoms (mean ± SD)	6 ± 2 months

**Table 2:** Clinical examination results.

Clinical Test	Positive (%)	Negative (%)
Tinel's sign	60 (60%)	40 (40%)
Phalen's maneuver	75 (75%)	25 (25%)

**Table 3:** Diagnostic accuracy metrics. *p<0.05.

Metric	Value
Sensitivity	75%
Specificity	60%
Positive Predictive Value (PPV)	70%
Negative Predictive Value (NPV)	65%
Chi-square (χ²)	4.02

The Chi-square test value was 4.02 with a p-value of 0.045, indicating a statistically significant association between clinical examination and NCS results (Table 3; p < 0.05). These findings suggest that although clinical examination is effective as an initial tool for diagnosing CTS, it lacks sufficient sensitivity and specificity when used alone.

The study also demonstrated that 70 patients had abnormalities in median nerve conduction velocity, and 65 patients had abnormalities in the amplitude of the median nerve during NCS (Table 4).

**Table 4:** Nerve conduction study (NCS) results.

NCS Parameter	Abnormal (%)	Normal (%)
Conduction velocity	70 (70%)	30 (30%)
Amplitude	65 (65%)	35 (35%)

The Chi-square test has been consistently used in the literature to correlate clinical signs with electrophysiological confirmation [10]. Similar proportions and diagnostic values have been reported in recent studies evaluating CTS diagnostic tools [11].

The results of this study support the view that clinical examination, together with NCS, should be used to facilitate the diagnosis and management of patients in clinical practice.

## Discussion

The clinical examination in diagnosing CTS is less effective when compared to NCS. The present study showed that clinical examination had a sensitivity of 75% and a specificity of 60%, indicating that although it is relatively accurate in detecting true positives, there is a considerable likelihood of false positives and false negatives. Furthermore, Makanji HS et al. [12], in their meta-analysis on clinical diagnostic tests for CTS, found comparable sensitivity and specificity values and concluded that confirmatory tests are required, supporting the findings of our study. Their analysis emphasized that NCS remains the gold standard because it provides quantitative data on nerve function, thereby minimizing diagnostic errors. Comparative research also supports that while USG and newer tools like high-resolution sonography and the Katz hand diagram show promise, they still lack the accuracy demonstrated by nerve conduction studies [13-15]. The PPV of 70% in our study suggests that there is a high probability of confirming CTS in patients clinically diagnosed when followed by a positive NCS result. This finding is particularly important in establishing the value of clinical examination in resource-poor settings where NCS may not be readily available. Nevertheless, the negative predictive value of 65% also indicates a notable risk of missing CTS diagnoses through clinical examination alone. Moreover, Atroshi I et al. [2] reported that CTS can be underdiagnosed by clinical examination, especially in its early or mild stages. Their study concluded that NCS can detect neurophysiological abnormalities not clinically observable, enabling earlier and more effective management of patients with neurological disorders. These findings are supported by Aseem F et al. [16], who found that even commonly used provocative tests like Tinel’s, Phalen’s, and Durkan’s had limited standalone diagnostic value. The p-value of 0.045 obtained in this study indicates a statistically significant relationship between clinical examination and NCS findings. This reinforces the importance of using NCS to confirm clinical suspicions, as also recommended by Rempel DM and Diao E [9], who noted that electrodiagnostic studies offer valuable insights that enhance diagnosis and guide treatment strategies. In clinical practice, a thorough clinical examination followed by NCS only when necessary provides a balanced approach. This strategy allows for efficient resource use while ensuring diagnostic accuracy. Katz JN and Stirrat CR [4] emphasized that combining clinical and electrodiagnostic methods improves diagnostic certainty and patient care. Additionally, differences in diagnostic accuracy across clinics and patient populations underscore the importance of training and clinical experience. Becker J et al. [17] demonstrated that clinicians with greater expertise in musculoskeletal examination achieved higher diagnostic accuracy, highlighting the need for continued education and training in CTS diagnosis. Finally, the results of the present study contribute to the growing body of literature supporting a multidimensional approach to diagnosing CTS. Integration of clinical examination with NCS enhances diagnostic yield and enables the development of individualized treatment plans, as noted by Werner RA and Andary M [18] and Bland JD [19]. In conclusion, while clinical examination is a valuable initial tool for detecting CTS, its limitations necessitate confirmation via NCS. This dual approach ensures accurate diagnosis and effective management, ultimately improving patient outcomes [20].

This study has a few limitations. The relatively small sample size, lack of blinding, non-random sample selection, and single-center setting may limit the generalizability of the findings. Variability in clinical examination techniques among clinicians, restricted NCS protocols, and limited demographic data may also impact the results. These limitations underscore the need for further research through larger, multi-center studies with more diverse samples, longitudinal designs, and exploration of high-resolution ultrasound as a cost-effective alternative or adjunct to nerve conduction studies in resource-limited settings.

## Conclusions

Clinical examination for diagnosing CTS is helpful; however, it lacks high sensitivity and specificity. Therefore, NCS should be used to confirm the diagnosis and ensure appropriate management of the patient’s condition. It is recommended that both techniques be used in combination to enhance diagnostic accuracy and improve patient outcomes. This study reinforces the importance of NCS in the assessment of CTS, confirming that it is a critical tool in accurately diagnosing the condition.
